# Analysis of the interests of Google users on toothache information

**DOI:** 10.1371/journal.pone.0186059

**Published:** 2017-10-19

**Authors:** Matheus Lotto, Patricia Estefania Ayala Aguirre, Daniela Rios, Maria Aparecida Andrade Moreira Machado, Agnes Fátima Pereira Cruvinel, Thiago Cruvinel

**Affiliations:** 1 Department of Pediatric Dentistry, Orthodontics and Public Health, Bauru School of Dentistry, University of São Paulo, Bauru, São Paulo, Brazil; 2 Department of Public Health, School of Medicine, Federal University of Fronteira Sul, Chapecó, Santa Catarina, Brazil; University of Washington, UNITED STATES

## Abstract

**Background:**

The knowledge on health interests of a given population of Internet users might contribute to the increase of evidence on community’s dental needs, and consequently, to the improvement of public health planning. The frequency of searches for specific issues on Google can be analyzed by the application of Google Trends.

**Aim:**

In this study, we aimed to characterize the interests on toothache information of Google users from the United States, United Kingdom, Australia and Brazil.

**Methods:**

The monthly variation of relative search volume (RSV) and the lists of main toothache-related queries were determined from January 2004 through April 2016 using Google Trends. Autoregressive Integrated Moving Average (ARIMA) forecasting models were developed to determine predictive RSV values for toothache for additional 12 months. Autocorrelation plots and general additive model (GAM) were applied to determine trends and seasonality in RSV curves. Through linear regression models, we assessed the association between the variation of annual means of RSV values and national statistics for toothache in the U.S. and U.K. Subsequently, the distribution of main queries according to the identification of endodontic pain, type of searching information, and the interest in self-management of toothache was evaluated for the four countries.

**Results:**

The autocorrelation plots showed patterns of non-stationary time series. The monthly variation influenced the data of the U.S. and U.K., with the higher RSV values found respectively in January/July and December. Also, the interest on toothache in the U.K. increases in the second semester and in the fourth quarter, especially in December. Additionally, an annual variation affected significantly all time series, with the increment of RSV means over the years, varying from 265% in the U.S. to 745% in Brazil. In parallel, the increments in RSV values were also observed in all predictive curves. The annual variation of observed and fitted RSV values was directly associated with the increase of toothache visits in the U.S. and urgent dental treatments in the U.K. Moreover, the queries typed on Google were markedly linked to searches on endodontic pain information, especially in Brazil, where the residents usually searched for relief and/or self-management of pain.

**Conclusions:**

Therefore, these findings indicate an increasing interest of Google users on toothache-related topics, regardless of country and season. The Internet activity can be used by policy makers as a complementary source of data for the development and implementation of strategies to control and prevent toothache complications.

## Introduction

The World Health Organization (WHO) and the World Dental Federation (FDI) recommended the reduction of toothache as one of the priority issues in the Global Oral Health Promotion Agenda [1]. Toothache is defined as an orofacial pain originated from a dental element and/or adjacent structures in consequence of several diseases or conditions, such as dental caries, periodontal disease, trauma, occlusal dysfunction, and abscess [2]. It is more prevalent among socioeconomic deprived groups [3, 4], affecting the sleep, feeding, school/work performance, and productivity [5, 6]; consequently, toothache negatively impacts the individuals’ quality of life [7]. For these reasons, people suffering with toothache may access the Internet searching for useful advices for the self-management of pain, such as medication, home remedies and emergency dental care [8]. Also, internet users navigate on the web to confirm professional instructions or to make self-diagnosis of alterations and/or diseases [9].

The health seekers frequently begin their web searches using the Google Search engine, which account for more than 70% of market share of this industry [10]. The Big Data produced from structured queries typed on Google can be systematically analyzed by Google Trends [11], an online tool developed to evaluate market and opinion trends on the Internet in near real-time. In this scenario, the awareness of the volume and profile of oral health searches performed on Google from specific regions might contribute to the recognition of community’s dental needs in order to guide policy makers in the development of further action plans, providing information to an area that traditional methods are largely incomplete: behavioral health [12], a multidisciplinary field that combines knowledge on health and human sciences with focus on the better understanding of health and disease [13]. It would be even more important when regarding the difficulty of obtaining accurate toothache data, since that diagnosis depends on how patients deal with physiological, observational and self-reporting components of pain [14]. In addition, access barriers prevent a sizeable proportion of individuals in reaching appropriate dental treatments, leading to under-registration of toothache [2, 15]. Recently, there has been an increasing use of the analysis of Internet activity to measure the people’s interest on medical conditions [16–20].

Taking into consideration the challenges posed by toothache, the aim of this study was to characterize the toothache-related interests of Google users from the United States, United Kingdom, Australia and Brazil. We hypothesize that the surveillance of health seeking behaviors of Internet users could provide complementary information about the impact of toothache in different population groups, aiding in the minimization of damage caused by the self-management of that clinical condition.

## Materials and methods

### Study design

This cross-sectional study was performed by the analysis of computational data of four distinct countries. After the development of specific query strategies, the relative search volume (RSV) and the popularity of toothache-related queries were determined using Google Trends, in two distinct periods. The RSV values indicate the ratio between the search volume of specific Google’s queries and the search volume of overall Google’s queries performed in specific regions and time intervals. These values are normalized in function of the maximum value of the time series (RSV = 100), varying from 0 to 100. Also, the main toothache-related terms typed on Google are ranked in descending order of their respective RSV values.

The data collected were assessed according to the following aspects: (a) validation of analysis, (b) search volume trends and seasonality, (c) production of forecasting models, (d) association with available toothache statistics, (e) relation with media interests, and (f) distribution of categories of main toothache-related topics.

### Countries selection

The countries were selected according to three criteria: (i) availability of more than 20 million Internet users, (ii) more than 50% of Internet penetration, and (iii) detection of relevant Internet-based toothache data in Google Trends. To evaluate season-based effects, two countries from each Hemisphere and distinct continents were chosen simultaneously. The United States, the United Kingdom, Australia, and Brazil presented all requirements for being included in this essay.

### Query strategies

**Fig 1:**
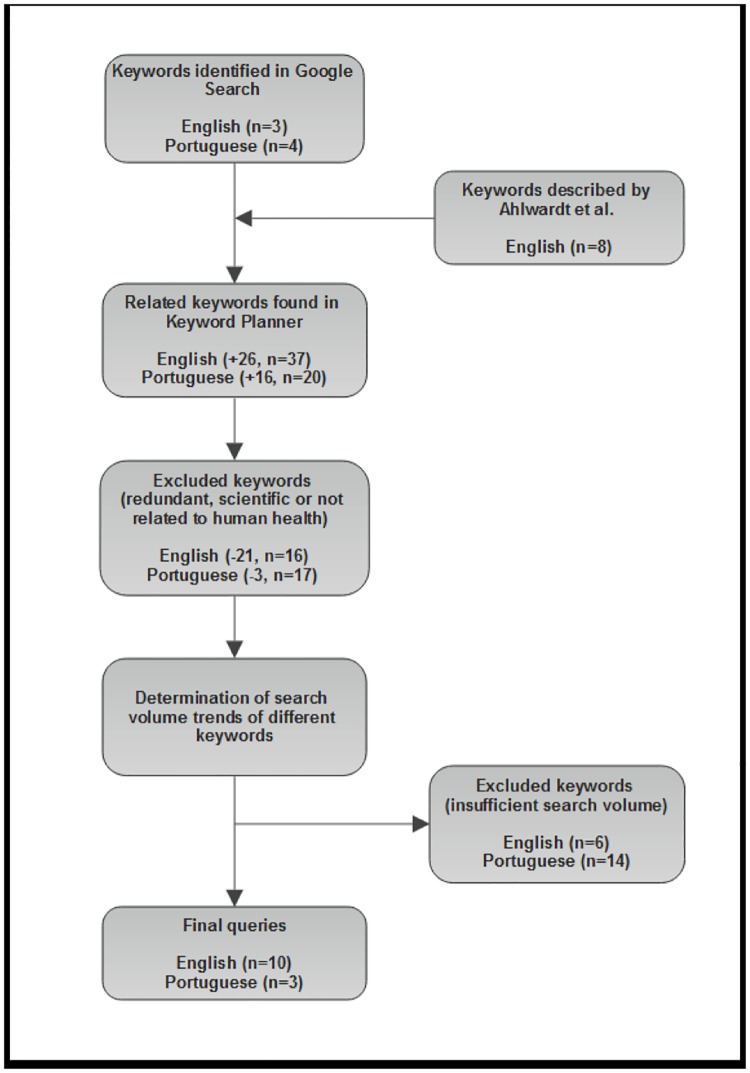
Flowchart summarizing the development of query strategies to assess the RSV toothache values in the U.S., U.K., Australia and Brazil.

The development of query strategies is shown in Fig 1. Initially, free queries written in English and Brazilian Portuguese were performed on Google Search to choose relevant toothache-related terms for the four selected countries. In this first step, only three terms in English [*“toothache”*, *“tooth pain”*, and *“aching tooth”*] and four terms in Portuguese [*“dor de dente”* (toothache), *“dor dente”* (toothache), *“dente dolorido”* (sore tooth), and *“dente doendo”* (aching tooth)] were considered relevant. Following, other eight toothache-related terms described by Ahlwardt et al [8] were added to the initial English term list.

Then, each term was individually inserted in *Keyword Planner* [21], a tool used to find new keywords based on pre-determined terms. After that, 26 terms in English and 16 terms in Portuguese were additionally included in the keyword lists; however, terms presenting redundant, scientific or not human-related characteristics were excluded (English -21, n = 16; Portuguese -3, n = 17). Finally, after the individual analysis of each term on Google Trends, keywords with irrelevant RSV values were also excluded (English -6, n = 10; Portuguese -14, n = 3).

The final query strategies are presented as follows: query #1 (English, applied to the analysis of the U.S., U.K. and Australia): [*"toothache"* + *"tooth pain"* + *"teeth hurt"* + *"sensitive tooth"* + *"sore teeth"* + *"sore tooth"* + *"tooth hurts"* + *"tooth sensitivity"* + *"throbbing tooth"* + *"dental pain"*]; query #2 (Brazilian Portuguese, applied to the analysis of Brazil): [*"dor de dente"* (toothache) + *"dor dente"* (toothache) + *"dente doi"* (tooth hurts)].

### Search volume trends

The RSV curves were obtained using Google Trends, as aforementioned. The data were collected specifically for each one of the four selected countries, in two distinct dates: November 2, 2014 and April 30, 2016. Both collections were performed on a monthly basis using all query categories, including results displayed since January 2004.

To confirm the equivalence of two query strategies in demonstrating the search volume of toothache-related terms in different countries, the trends related to the topics “Toothache—Disease”, a set of automatic algorithms supplied by Google Trends, were also achieved for the U.S., U.K., and Brazil in the second session of data collection (April 30, 2016). Google Trends did not provide automatic algorithms for Australia.

### Main queries

The main toothache-related queries used by the Internet users from different countries were saved in.*csv* files, also provided by the platform of Google Trends. An investigator dichotomized those queries according to three categories: identification of endodontic pain (no/yes), type of searching information (cause-symptoms/relief-treatment), and interest in self-management strategies of toothache (no/yes). “No” and “cause-symptoms” were coded as 0 (zero), while “yes” and “relief-treatment” were coded as 1 (one). Each query was weighted by its respective RSV value. The differences in the distribution of toothache characteristics in distinct countries were determined for each category by the comparison of the percentage of weighted queries.

### Available statistics for toothache

The U.S. toothache statistics and population estimates between the years 2005 and 2010 were respectively extracted from *The National Hospital Ambulatory Medical Care Survey* (NHAMCS) [22] and the U.S. Census Bureau [23] databases. The variables age, sex, patient’s reason for visit (*rfv1-3*), and patient visit weight (*patwt*) were considered to calculate the absolute and relative number of toothache visits registered by the Emergency Department. The weights of patients *(patwt)* with diagnosis of toothache were summed to obtain the amount of toothache cases per 1,000 population, per age group (15–69 y, *x1*,*000 pop*.), and per female sex (*x1*,*000 pop*.) on an annual basis.

The estimates of the number of urgent dental treatments in England per 1,000 population and adult age group were based on the official reports of the NHS Dental Statistics for England [24]. The population data were obtained from the Office for National Statistics [25]. Both statistics were published between 2006/2007 and 2014/2015.

The toothache statistics of Australia were obtained from the *Australian Institute of Health and Welfare* [26], which provided a curve trend of toothache cases among persons aged 15 and over, from 1994 through 2010. The prevalences of toothache in Brazil were collected from the reports of two distinct surveys–*SB Brasil 2003* [27] and *SB Brasil 2010* [28].

### Data analysis

Data were analyzed with the Statistical Package for Social Science (version 21.0; SPSS, Chicago, USA), considering the following aspects:

1. Validation of analysis: The Intraclass Correlation Coefficient (ICC) was used to assess the stability of RSV toothache data collected in two dates (Nov 2014 and Apr 2016), and to analyze the absolute concordance between the curves originated from the present query strategies and those curves originated from automatic algorithms by Google Trends. The predictive performance of preliminary forecasting models (November 2014) was evaluated based on real data collected in April 2016 (see details on predictive analysis below).
2. Search volume trends and seasonality: The autocorrelation and partial autocorrelation plots of the RSV toothache values were analyzed to identify the patterns of fluctuation of data over time. The effect of seasonality on the time series was evaluated by generalized additive model (GAM). It was comprised by a previous detrending of each long-term curve by its lag-1 difference, with subsequent application of distinct generalized linear models to evaluate the effect of monthly, quarterly, semi-annual and annual seasonality on time series.
3. Production of forecasting models: The data collected in April 2016 were used to construct 12-month forecasts for RSV toothache values. For this purpose, Autoregressive Integrated Moving Average (ARIMA) models were chosen by the lowest values of Normalized Bayesian Information Criteria (Normalized BIC), Root Mean Square Error (RMSE), and Mean Absolute Percentage Error (MAPE), among those curves without significant residual autocorrelation (Ljung-Box test, *P*>0.05).
4. Association with available toothache statistics: The association of the annual mean variation of observed and fitted RSV toothache values with annual statistics for toothache (U.S.) or urgent dental visits (U.K.) were determined by linear regression models. The missing value of the number of urgent dental treatments in England in 2007/2008 was previously replaced by linear interpolation. Due to the absence of official annual statistics, these analyses were not performed for Australia and Brazil.
5. Relation with media interests: The months associated with the abrupt RSV spikes, identified as outliers in ARIMA models, were qualitatively analyzed in relation to toothache-related media publications. Seven types of outliers were considered for this analysis: additive, level shift, innovational, transient, seasonal additive, local trend, and additive patch.
6. Distribution of categories of main toothache-related topics: The differences in the distribution of the main toothache-related topics between distinct countries were evaluated by Chi-square Pearson test.

For all statistical analyses, *P* values < 0.05 were considered significant.

## Results

### Validation of analysis

The RSV data of the four countries demonstrated an excellent stability over time. The minimum and maximum ICC values for consistency were respectively found in Brazil (0.88, 95% CI:0.82–0.91) and U.S. (0.99, 95% CI:0.99–1.00). Also, the absolute concordance between the current RSV toothache and the Google’s automatic time series varied from 0.95 (U.K., 95% CI:0.49–0.99) to 0.98 (Brazil, 95% CI:0.98–0.98).

The preliminary 12-month forecasts displayed significant increments of RSV toothache values in the U.S. (6.04%), U.K. (4.56%), Australia (3.26%) and Brazil (7.10%). These results were fully confirmed by real data collected in April 2016; nevertheless, the forecasts were more conservative in the U.K., Australia and Brazil, with respective gains of 8.89%, 5.36%, and 26.23%. On the other hand, the U.S. showed a lower variation (3.72%) than that predicted.

### Search volume trends and seasonality

**Fig 2:**
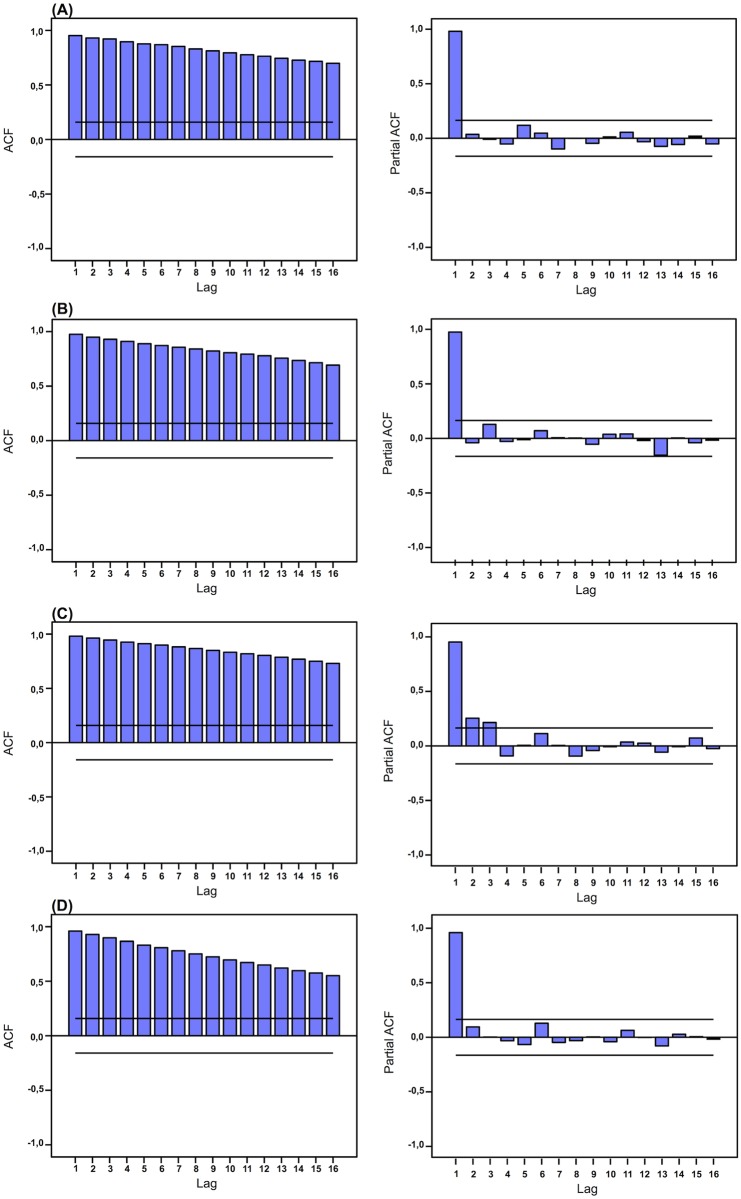
Autocorrelation and partial autocorrelation plots for the monthly variation of RSV toothache values. (A) U.S., (B) U.K., (C) Australia, and (D) Brazil.

**Fig 3:**
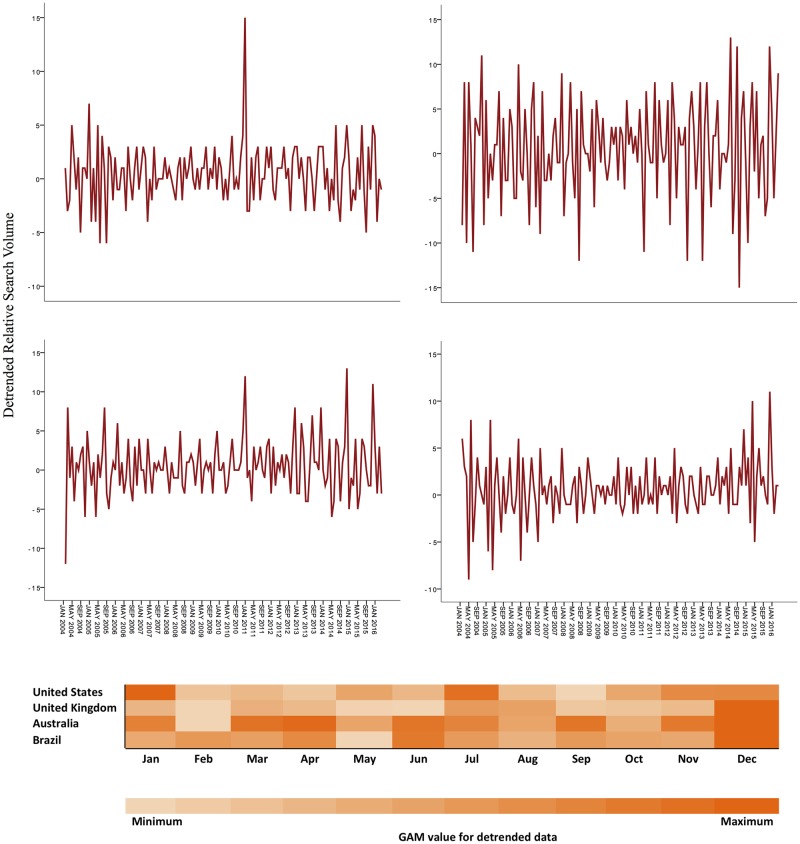
Seasonality of the interests of Google users on toothache. It was estimated by fitting a generalized additive model (GAM) to the detrended Google Trends data (lag-1 difference). Note that the scales vary from negative to positive values, since they represent the variation of values of the RSV of a month minus the RSV of the previous month. For instance, the detrended value of December 2004 is represented by the difference of its RSV in relation to the RSV of November 2004. GAM values using monthly RSV as a predictive variable for Google data are represented in the heat map.

The autocorrelation plots clearly demonstrate the pattern of non-stationary time series (Fig 2). The monthly variation influenced the data of the U.S. and U.K., with the higher RSV values found respectively in January/July and December (Fig 3). Also, the interest on toothache in the U.K. increases in the second semester and in the fourth quarter, especially in December (Fig 3). Additionally, an annual seasonality affected significantly all time series, with considerable increments of RSV means over the years, varying from 265% in the U.S. to 745% in Brazil (Figs 2 and 3).

### Forecasting models

**Fig 4:**
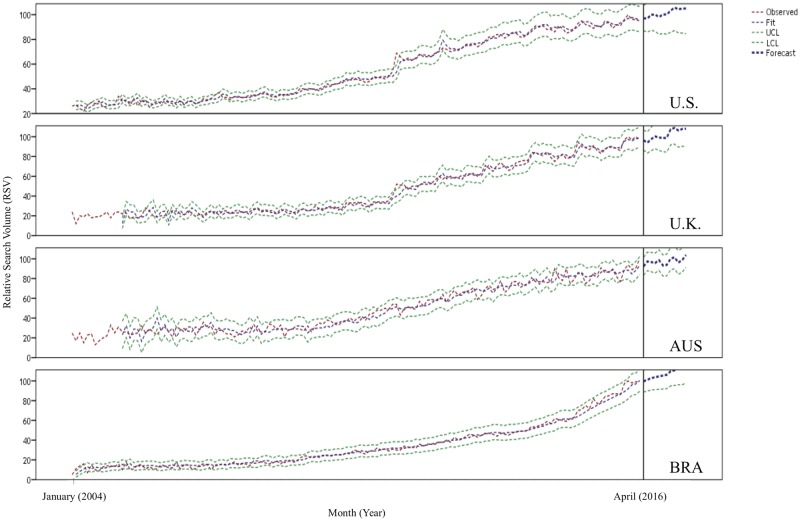
Predictive charts demonstrate the variation of RSV toothache values in the U.S., U.K., Australia and Brazil from January 2004 through April 2017. Note that RSVs presented after April 2016 (black line) represent 12-month predictive values. Observe the increase of the interest of Google users on toothache in all countries.

Tables 1 and 2 summarize the adequacy measures and parameter estimation of forecasting models for RSV toothache values collected in April 2016. The chosen ARIMA models demonstrated the lowest Normalized BIC (1.88–3.23), RMSE (2.43–4.85), and MAPE (3.65–9.57) values, without detection of residual autocorrelation (Ljung-Box, *P*>0.05). The curves of observed and fitted RSV values of the four countries presented a trend of increase over time (Fig 4).

**Table 1:** ARIMA model fit statistics.

					Ljung-Box	
Country, Model	*R*^*2*^	Normalized BIC*	RMSE^†^	MAPE^‡^	Statistics	*P*
United States **ARIMA (0,1,1)(1,0,0)**	0.99	1.88	2.43	3.65	18.17	0.31
United Kingdom **ARIMA (0,1,1)(0,1,1)**	0.98	2.51	3.38	7.21	23.48	0.10
Australia **ARIMA (0,1,1)(0,1,1)**	0.96	3.23	4.85	9.57	13.74	0.62
Brazil **ARIMA (0,1,1)(1,0,1)**	0.99	2.12	2.70	9.47	22.06	0.11

* Normalized Bayesian Information Criteria

^†^ Root mean square error

^‡^ Mean absolute percentage error

**Table 2:** Parameter estimation of ARIMA models for RSV toothache values in distinct countries.

			Estimate	SE*	t	*P*
**United States**	Constant		0.01	0.004	2.32	0.02
	Difference		1			
	MA^†^	Lag 1	0.48	0.07	6.53	<0.001
	AR^‡^, Seasonal	Lag 1	0.46	0.07	6.24	<0.001
**United Kingdom**	Difference		1			
	MA	Lag 1	0.56	0.07	7.59	<0.001
	Seasonal Difference		1			
	MA, Seasonal	Lag 1	0.90	0.14	6.37	<0.001
**Australia**	Difference		1			
	MA	Lag 1	0.72	0.06	11.25	<0.001
	Seasonal Difference		1			
	MA, Seasonal	Lag 1	0.74	0.09	8.05	<0.001
**Brazil**	Constant		0.05	0.01	4.43	<0.001
	Difference		1			
	MA	Lag 1	0.68	0.07	10.50	<0.001
	AR, Seasonal	Lag 1	0.82	0.20	4.16	<0.001
	MA, Seasonal	Lag 1	0.70	0.25	2.83	0.01

* Standard error

^†^ Moving average component

^‡^ Autoregressive component

### Association with available toothache statistics

Table 3 depicts a positive significant association between the variation of the annual means of observed and fitted RSV toothache values and the increment of toothache visits in the U.S. (+29.7%) or urgent dental treatments in the U.K. (+21.9%). The toothache statistics presented for the total population of the U.S. and adult population of the U.S. and U.K. were stronger correlated with RSV toothache values than other population groups.

**Table 3:** Association of the variation of annual means of observed and fitted RSV toothache values with national statistics of toothache (U.S.) or urgent dental treatments (U.K.).

	B*	95% CI^†^	β	*R*^*2*^	*P*
*Observed values*					
**United States**					
Toothache visits (total, per *1*,*000 pop*.)	14.12	6.59–21.64	0.93	0.87	0.006
Toothache visits per age (adults, per *1*,*000 pop*.)	10.93	6.17–15.68	0.95	0.91	0.003
Toothache visits per gender (female, per *1*,*000 pop*.)	12.78	3.80–21.77	0.89	0.80	0.017
**United Kingdom**					
Urgent dental treatments (total, per *1*,*000 pop*.)	3.48	0.60–6.37	0.77	0.59	0.026
Urgent dental treatments per age (adults, per *1*,*000 pop*.)	3.43	0.78–6.07	0.79	0.63	0.019
*Fitted values*					
**United States**					
Toothache visits (total, per *1*,*000 pop*.)	14.02	6.90–21.14	0.94	0.88	0.005
Toothache visits per age (adults, per *1*,*000 pop*.)	8.20	3.43–12.97	0.92	0.85	0.009
Toothache visits per gender (female, per *1*,*000 pop*.)	12.78	4.34–21.22	0.90	0.82	0.014
**United Kingdom**					
Urgent dental treatments (total, per *1*,*000 pop*.)	2.95	0.49–5.40	0.77	0.59	0.026
Urgent dental treatments per age (adults, per *1*,*000 pop*.)	2.90	0.65–5.15	0.79	0.62	0.020

* Unstandardized coefficient

^†^ 95% confidence interval

### Relation with media interests

Twenty-seven outliers were detected in different time series, which were distributed among the U.S. (n = 10), U.K. (n = 5), and Brazil (n = 12). Only a scientific article published in January 2005 was connected to an additive patch outlier in the U.S. time series. Therefore, the media interests did not disturb the variation of RSV toothache values in any of the four countries.

### Main topics of toothache information

Although the main toothache queries were markedly associated with searches on endodontic pain information, the proportion found in Brazil was still significantly higher than other countries. For instance, queries as “earache”, “wisdom tooth”, “sinus toothache”, “sinus infection”, “sensitive tooth”, and “jaw pain” were categorized as non-endodontic pain. In addition, Brazilian residents seemed to be more prone to seek instructions about relief/treatment of toothache compared to other nationalities. The adoption of self-management strategies, such as the consumption of medications and home remedies, were usually identified among individuals interested in relief/treatment pain, mainly in the U.S. and Brazil (Table 4). The percentages of searches related to adequate treatment of toothache were 1.2% in Brazil, 1.8% in the U.S., 3.9% in Australia, and 7.9% in the U. K. (Table 5).

**Table 4:** Toothache-related queries and their respective RSV for each country. Terms with RSV = 0 were not considered.

U.S.		U.K.		Australia		Brazil	
toothache remedies	100	toothache pain	100	toothache pain	100	toothache remedies	100
toothache pain	100	wisdom tooth	95	wisdom tooth	85	remedies	100
home remedies	65	wisdom tooth pain	90	wisdom tooth pain	75	painkiller	75
wisdom tooth	60	toothache relief	50	toothache relief	55	home remedies	25
my teeth hurt	55	toothache remedies	45	toothache remedies	45	dental pain	20
toothache remedy	55	toothache cure	35	wisdom teeth	40	remedies for toothache	20
wisdom tooth pain	55	wisdom teeth	35	toothache remedy	40	root canal	15
home toothache remedies	50	my teeth hurt	30	my teeth hurt	35	root canal pain	15
teeth pain	50	toothache pain relief	25	toothache pain relief	30	pregnant toothache	10
remedies for toothache	50	root canal	25	tooth pain relief	25	teeth pain	10
toothache relief	40	abscess	25	root canal	25	tooth inflammation	5
root canal	40	emergency dentist	25	tooth ache	25	root canal hurts	5
wisdom teeth	35	stop toothache	20	stop toothache	25	antiinflammatory	5
tooth infection	35	tooth pain relief	20	cloves	25	earache	5
tooth pain relief	30	tooth ache	20	remedies for toothache	20	wisdom tooth	5
remedy for toothache	25	clove	20	toothache cloves	20	wisdom teeth	5
sinus infection	25	wisdom teeth pain	20	wisdom teeth pain	20	wisdom tooth pain	5
home remedy toothache	25	toothache remedy	20	sinus pain	15	root canal treatment	5
stop toothache	25	tooth infection	20	home remedies toothache	15	antiinflammatory for tooth	5
my tooth hurts	25	tooth abscess	20	tooth infection	15	analgesic	5
tooth pain remedies	25	clove oil	20	toothache symptoms	15	toothache ICD	5
toothache help	25	relief for toothache	20	tooth abscess	15	endodontic treatment	5
root canal pain	25	clove oil toothache	15	my tooth hurts	15		
sinus tooth pain	25	severe toothache	15	cure toothache	15		
cavity	20	bad toothache	15	clove oil	15		
tooth ache	20	remedies for toothache	15	toothache sinus	15		
jaw pain	20	toothache symptoms	15	toothache cures	15		
toothache medicine	20	toothache headache	15	remedy for toothache	15		
sinus toothache	15	toothache treatment	15	wisdom teeth hurt	15		
bad toothache	15	tooth extraction	15	toothache causes	15		
wisdom teeth pain	15	toothache home remedies	15	toothache treatment	10		
toothache cure	15	cure for toothache	10	clove oil toothache	10		
clove oil	15	sinus toothache	10	cloves for toothache	10		
tooth abscess	15	my tooth hurts	10	sinus tooth pain	10		
toothache pain relief	15	painkillers for toothache	10	bad toothache	10		
severe tooth pain	15	toothache causes	10	toothache pregnancy	10		
pregnant toothache	15	toothache after filling	10	root canal pain	10		
clove oil toothache	15	painkiller for toothache	10	toothache headache	10		
severe toothache	15	toothache cures	10	tooth nerve pain	10		
tooth pain remedy	10	i have toothache	10	sensitive teeth	10		
toothache symptoms	10	oil for toothache	10	sinus infection	10		
toothache headache	10	root canal pain	10	toothache home remedy	10		
stop tooth pain	10	sensitive teeth	10	oil of cloves	10		
teeth hurt sinus	10	dental pain relief	10	relieve toothache	10		
relieve tooth pain	10	sinus infection	10				
toothache causes	10	cloves toothache	10				
medicine for toothache	10						
relief for toothache	10						

**Table 5:** Distribution of the main toothache-related queries according to distinct categories.

**(A)**	**Identification of endodontic pain**	
	**No** **n (%)**	**Yes** **n (%)**
United States^a^	360 (26.1%)	1020 (73.9%)
United Kingdom^b^	330 (37.3%)	555 (62.7%)
Australia^b^	305 (39.6%)	465 (60.4%)
Brazil^c^	15 (3.7%)	395 (96.3%)
Chi-square (value, *P*)	211.77	<0.001
**(B)**	**Type of searching information**	
	**Causes/Symptoms** **n (%)**	**Relief/Treatment** **n (%)**
United States^a^	740 (53.6%)	640 (46.3%)
United Kingdom^b^	575 (65.0%)	310 (35.0%)
Australia^b^	485 (63.0%)	285 (37.0%)
Brazil^c^	100 (24.4%)	310 (75.6%)
Chi-square (value, *P*)	203.71	<0.001
**(C)**	**Interest in self-management strategies for toothache**	
	**No** **n (%)**	**Yes** **n (%)**
United States^a^	25 (3.9%)	615 (96.1%)
United Kingdom^b^	70 (22.6%)	240 (77.4%)
Australia^c^	30 (10.5%)	255 (89.5%)
Brazil^a^	5 (1.6%)	305 (98.4%)
Chi-square (value, *P*)	117.87	<0.001

Different lower-case superscript letters indicate significant difference between two distinct countries (Chi-square, *P*<0.05).

## Discussion

These findings indicate a continuous increase of the interests of the Internet users on toothache information over the years. These trends are consistent among different countries, without influence of media. In most cases, the Internet is used as a source of instruction on how to self manage dental pain, through the consumption of medication and/or home remedies. To our knowledge, this is the first study that demonstrates the utility of Google-based data to improve the understanding of dental needs of distinct countries.

The search volume trends on toothache are in agreement with the national statistics of the U.S., U.K., and Australia, which registered the increase of the incidence of toothache along time [22, 24, 26]. Honkala et al [29] have displayed a growth of toothache cases among Finnish adolescents from 1977 to 1997, even with the decline of dental caries. More recently, Lewis et al [30] found a gradual increase in toothache cases among U.S. young adults who sought emergency dental treatments in the 2000’s decade. Marcenes et al [31] demonstrated that the burden of untreated dental caries in permanent teeth raised 3.2% from 1990 through 2010. The impact of tooth loss among children and adolescents progressively decreased in response to the reduction of dental caries rates. This fact in conjunction with the population aging may explain the increase of untreated caries lesions among adults, and by consequence, the higher incidence of toothache [32]. The deterioration of the employability and the health systems due to the financial crises hampers the access of individuals to preventive dental visits, also contributing to the increment of toothache [29, 33]. In this sense, Althouse et al [20] showed the increment of toothache concerns of web health seekers during the U.S. Great Recession (2008–2011). Other seasonal stressor might explain the significant increase of toothache-related queries in December and January: Christmas festivities. The impact of Christmas ranges from increased stress, family conflicts and alcohol misuse to heightened loneliness, increasing mental health difficulties and domestic violence [34]. Curiously, the higher means of detrended RSV values for toothache were also observed during December in Brazil and Australia.

Although our results demonstrate a trend of increase of search volume of toothache-related queries in Brazil, the national statistics produced in 2003 and 2010 revealed a substantial decline of toothache cases, from 34.8% to 27.5% [27, 28]. This divergent result might be explained by the advancement of digital penetration, which facilitates the access of deprived groups to the Internet, typically more affected by toothache [35, 36]. This greater Internet access also intensifies the health seeking behavior, leading persons to begin their toothache-related searches when are experiencing milder pain [29]. Although this overreaction to pain might be a confounding factor for toothache analysis, a deeper overview of the present queries elucidated the endodontic pain as the main cause of Internet toothache searches in Brazil. On the other hand, two methodological differences found between the Brazilian surveys might influence the interpretation of these results. The sampling criteria adopted in the surveys differed significantly from each other, with the preponderant inclusion of metropolitan areas in the second survey. The greater availability of healthcare services in urbanized areas may facilitate the access to dental treatment, reducing the number of toothache cases among those population groups. Besides, in both surveys, the prevalence of toothache was calculated through the recall of participants about their last 6-month dental experiences, which predisposes to inaccurate reports and observer-expectancy effect [37, 38]. Differently, the prevalence of toothache in the U.S., U.K. and Australia was based on the information collected from the emergency patient records [22, 24, 26].

It is not possible to affirm that all queries typed on Google were performed by people experiencing toothache; however, the no interference of media on the behavior of time series and the direct link of main queries with the self-management of endodontic pain indicate that searches were mostly conducted by individuals interested in the resolution of dental pain. Also, the overestimation of the interest on toothache cannot be discarded due to possible duplicate searches, since Google accounts all specific queries originated from different Internet Protocols (IPs) in a time range [11]. In parallel, Santillana et al. [39] exhibited the tendency of Google Flu Trends in overestimating cases of influenza over time. Indeed, a same person suffering with toothache can search on Google from diverse computers, localized at home, work and public places. While this repetitive action limits the potential of Internet-based methods for replacing traditional epidemiological approaches, the registration of multiple queries from a single person may indirectly represent the severity of pain, the importance of health barriers or even the time elapsed until the dental visit; therefore, this analysis can contribute greatly with the elucidation of behavioral aspects related to toothache. It is noteworthy that the number of people that used Google for seeking adequate treatment of toothache was alarmingly low, varying from 1.2% in Brazil to 7.9% in the U.K. These results are even more dramatic when the profile of Google health seekers is considered, composed predominantly by white women with high income and education [9].

The individuals interested in relief and/or treatment of toothache usually searched for self-management of pain, through the utilization of home remedies and/or over-the-counter painkillers. These results corroborate with previous social media-based findings [8, 40]. Also, a major impact of the self-management of endodontic pain was found in Brazil, a developing country with profound social inequalities and, therefore, with less access to dental treatment. These results bring up the concern about the negative effect of incorrect and/or inaccurate contents of websites on the deterioration of the patient’s health conditions, hampering the person-professional relationship and the shared decision-making process.

The abundance of Google Trends’ data enables the development of good fitted forecasting models for the prediction of health interests of specific populations in a near future, overcoming the expected delay for the production and publication of dental statistics. Furthermore, this approach presents other advantages: i) the anonymous and objective collection of data decreases the reporting bias of surveys; ii) the analysis can be periodically updated and filtered for particular areas; iii) the assessment of the consumption of health information is simpler, faster, and more economical than interview-based methodologies [19]; iv) it provides near real-time data [37, 38]; v) the methodological standardization enables the comparison of results from distinct studies; vi) these data can be useful to improve the quality of surveys, by the inclusion of relevant questions and identification of new hypotheses using online proxies [12]. It should also be considered that the greater access to mobile technologies as smart phones and tablets, and the diffusion of high-speed Internet lead to the increment of the number of health-related queries in the next years. Additionally, the Internet penetration is advancing consistently even among the poorest countries [41], which substantiate the representativeness of samples in further studies.

The strength of these methods can be observed through the excellent stability of time series data, with the maintenance or improvement of the association level between the national statistics for toothache and the fitted RSV curves, which indicates a low risk of spurious correlation. In this analysis, we had to compare the RSV toothache values obtained for U.K. with dental statistics of England due to the lack of respective data. In our opinion, this comparison is feasible because the RSV data for England is presented as a constant value of the U.K. time series. Moreover, the confirmation of all preliminary forecasts demonstrated the power of ARIMA models in predicting the direction of persons’ interests on toothache information. Likewise, the strong positive correlation between the curves resulting from the English or Portuguese query strategies with those curves given by automatic Google Trends’ algorithms revealed the potential for comparison of results produced through distinct languages.

In conclusion, Google users from the U.S., U.K., Australia and Brazil demonstrated an increasing interest for toothache information, despite of media influence. Based on these results, the Internet activity can be used as a complementary source of data to support policy health makers in the development and implementation of person-centered strategies, such as the awareness campaigns on the possible consequences of untreated toothaches and/or the training of professional teams to prescribe good quality dental information for their patients. These measures would be relevant for minimizing the damage caused by the non-effective self-management of toothache.
